# Draft Genome Sequence of *Erwinia dacicola*, a Dominant Endosymbiont of Olive Flies

**DOI:** 10.1128/MRA.01067-18

**Published:** 2018-09-13

**Authors:** Anne M. Estes, David J. Hearn, Suvarna Nadendla, Elizabeth A. Pierson, Julie C. Dunning Hotopp

**Affiliations:** a Institute for Genome Sciences, University of Maryland School of Medicine, Baltimore, Maryland, USA; b Department of Biological Sciences, Towson University, Towson, Maryland, USA; c Department of Horticultural Sciences, Texas A&M University, College Station, Texas, USA; d Department of Microbiology and Immunology, University of Maryland School of Medicine, Baltimore, Maryland, USA; Indiana University Bloomington

## Abstract

Erwinia dacicola is a dominant endosymbiont of the pestiferous olive fly. Its genome is similar in size and GC content to those of free-living *Erwinia* species, including the plant pathogen Erwinia amylovora. The E. dacicola genome encodes the metabolic capability to supplement and detoxify the olive fly’s diet in larval and adult stages.

## Abstract (precis)

Erwinia dacicola is a dominant endosymbiont of the pestiferous olive fly. Its genome is similar in size and GC content to those of free-living *Erwinia* species, including the plant pathogen Erwinia amylovora.

## ANNOUNCEMENT

Erwinia dacicola Oroville was sequenced to determine its potential beneficial role in supplementing the diet of the tephritid olive fly, Bacterocera oleae. E. dacicola is the dominant bacterium that resides in specialized digestive system structures within the olive fly (1, 2) and is found in the vast majority of wild olive flies sampled from Greece, Italy, and the United States (3, 4). Most laboratory olive fly populations reared on artificial diets are not colonized by E. dacicola (3) and are not as healthy as wild olive flies (3, 5). To date, a draft E. dacicola genome sequence (6) and transcriptome (7) are available from populations of olive flies in Greece. European and U.S. olive flies belong to haplotypes htA and htB, respectively (4). The U.S. population most likely was founded by European olive flies, providing an interesting population for comparison.

E. dacicola has not been successfully cultured from the olive fly (1, 7, 8); therefore, DNA for the E. dacicola genome was isolated from four sets of 6 to 10 sterilely isolated olive fly esophageal bulbs. The bulbs were homogenized, and the bacteria were separated from host tissues using centrifugation. DNA was extracted using the DNeasy (Qiagen) Gram-negative protocol, and the 16S rRNA was amplified, cloned, and sequenced to verify that the DNA was solely from E. dacicola without contaminants. DNA was subsequently amplified using an Illustra GenomiPhi version 2 kit, and ∼8 µg was used to construct tagged, multiplexed libraries for sequencing on an Illumina Genome Analyzer II at the Arizona Genomics Institute at the University of Arizona, generating a total of 25,876,346 paired 75-bp reads. Except where otherwise specified, default parameters were used for all software. Sequences were assembled into contigs using *de novo* assembly in ABySS-pe version 1.2.5 with 4 different kmer sizes (40, 45, 50, 55) with a minimum contig length of 300 bp. The K55 assembly was selected since it had a genome size closest to that of the genome of E. amylovora and is the most complete E. dacicola genome sequenced to date. A total of 1,039 contigs spanning 2,858,157 bp were produced with an *N*_50_ value of 5,472 bp, maximum scaffold size of 79 kbp, 52.2% GC content, and >300× sequencing depth. The Institute for Genome Sciences (IGS) Annotation Engine (9) using Glimmer version 3.02 (10) identified 4,033 open reading frames (ORFs). Both the genome size and GC content are more similar to those of free-living bacteria than those of other intracellular bacteria found in other insects.

The genome encodes the ability to supplement amino acids and vitamins missing from the olive fruit on which the larvae feed. Similar to other draft genomes, the encoded potential of E. dacicola suggests that this endosymbiont may supplement and detoxify the fly diet.

### Data availability.

The reads (Sequence Read Archive number SRP155530), assembly, and annotation can be accessed through BioProject number PRJNA288714 and BioSample number SAMN03836967. This whole-genome shotgun project has been deposited at DDBJ/ENA/GenBank under the accession number LJAM00000000. The version described in this paper is version LJAM02000000.
